# COVID-19 Vaccine Acceptance and Hesitancy Among Health Care Workers in Lebanon

**DOI:** 10.1007/s44197-023-00086-4

**Published:** 2023-02-03

**Authors:** Nour J. Youssef, Nadim K. Tfaily, Mohammad Bahij M. Moumneh, Celina F. Boutros, Jad A. Elharake, Amyn A. Malik, SarahAnn M. McFadden, Bayan Galal, Inci Yildirim, Kaveh Khoshnood, Saad B. Omer, Ziad A. Memish, Ghassan S. Dbaibo

**Affiliations:** 1 Center for Infectious Diseases Research, Division of Pediatric Infectious Diseases, Department of Pediatrics and Adolescent Medicine, American University of Beirut Medical Center, Hamra , 1107 Beirut Lebanon; 2 Division of Pediatric Infectious Diseases, Department of Pediatrics and Adolescent Medicine, American University of Beirut Medical Center, Hamra, 1107 Beirut Lebanon; 3 Yale Institute for Global Health, New Haven, CT 06510 USA; 4 Department of Internal Medicine, Infectious Disease, Yale School of Medicine, New Haven, CT 06510 USA; 5 Yale University, New Haven, CT 06520 USA; 6 Department of Epidemiology of Microbial Diseases, Yale School of Public Health, New Haven, CT 06510 USA; 7 Yale School of Nursing, Orange, CT 06477 USA; 8 Research and Innovation Center, King Saud Medical City, Ministry of Health, Riyadh, Kingdom of Saudi Arabia; 9 College of Medicine, Al Faisal University, Riyadh, Kingdom of Saudi Arabia; 10 Hubert Department of Global Health, Rollins School of Public Health, Emory University, Atlanta, GA USA

**Keywords:** Health care workers, COVID-19, Lebanon, Vaccine acceptance, Refusal, trust

## Abstract

**Background:**

Lebanon endured its worst economic and financial crisis in 2020–2021. To minimize the impact of COVID-19 pandemic, it is important to improve the overall COVID-19 vaccination rate. Given that vaccine hesitancy among health care workers (HCWs) affects the general population’s decision to be vaccinated, our study assessed COVID-19 vaccine acceptance among Lebanon HCWs and identified barriers, demographic differences, and the most trusted sources of COVID-19 information.

**Methods:**

A cross-sectional study was conducted between January and May 2021 among HCWs across nine hospitals, the Orders of Physicians, Nurses, and Pharmacists in Lebanon. Descriptive statistics were performed to evaluate the COVID-19 vaccine acceptance, and univariate and multivariable to identify their predictors.

**Results:**

Among 879 participants, 762 (86.8%) were willing to receive the COVID-19 vaccine, 52 (5.9%) refused, and 64 (7.3%) were undecided. Males (226/254; 88.9%) and those ≥ 55 years (95/100; 95%) had the highest rates of acceptance. Of the 113 who were not willing to receive the vaccine, 54.9% reported that the vaccine was not studied well enough. Participants with a previous SARS-CoV-2 infection and those who did not know if they had a previous infection (*p* = 0.002) were less likely to accept the vaccine compared to those with no previous infection. The most trusted COVID-19 sources of information were WHO (69.3%) and healthcare providers (68%).

**Conclusion:**

Lebanese HCWs had a relatively high acceptance rate for COVID-19 vaccination compared to other countries. Our findings are important in informing the Lebanese health care authorities to establish programs and interventions to improve vaccine uptake among HCWs and the general population.

**Supplementary Information:**

The online version contains supplementary material available at 10.1007/s44197-023-00086-4.

## Introduction

The coronavirus disease-2019 (COVID-19) pandemic has resulted in more than 500 million confirmed cases and over 6.2 million deaths (as of May 2022) [1]. While the COVID-19 pandemic has had detrimental health, cultural, and socioeconomic effects across all countries, low- and middle-income countries (LMICs), such as Lebanon, are at greater risk of infection [2]. The first case of COVID-19 in Lebanon was confirmed on February 21st, 2020, and since then, the number of cases and deaths has significantly grown [3, 4].

Enormous international efforts have been put in the development and distribution of COVID-19 vaccines. The COVID-19 Vaccines Global Access Facility (COVAX) was launched [5], in collaboration with the World Health Organization (WHO), with a goal of administering at least two billion COVID-19 vaccine doses across LMICs by 2022 [6–8]. In Lebanon, the Ministry of Public Health (MOPH) launched its vaccination rollout on February 14th, 2021, starting with the Pfizer-BioNTech mRNA vaccine. An additional 1.5 million doses were reserved from Oxford-AstraZeneca to speed up the distribution of vaccines [7]. However, the success of immunization campaigns depends on acceptance of newly introduced vaccines, particularly during pandemics [9]. Vaccine hesitancy remains a pertinent issue across the general populations as well as among health care workers (HCWs), globally. Vaccine acceptance is known to vary with time and context [10]. Therefore, vaccine acceptance of a newly developed vaccines, especially as novel technology (e.g., mRNA vaccines) is being implemented, has historically been a concern [11].

Due to the limited vaccine supply across the world, priority was given to HCWs and other front-line workers to be immunized first. One of the major concerns was the level of vaccine acceptance across these HCWs [12]. An international survey on COVID-19 vaccine acceptance, conducted from June 16 to June 20, 2020, showed that at least 30% would be hesitant to take the vaccine [11]. A survey conducted in April 2020 in the United States (US) estimated refusal of COVID-19 vaccination in at least one third of the participants [13]. A national survey conducted between October and December 2020 in the Kingdom of Saudi Arabia (KSA) showed that the acceptance of COVID-19 vaccine among HCWs was around 65%, with the main reason for hesitance being fear of possible side effects [14]. In addition to several other vaccine acceptance studies [15–20], the KSA study highlighted the need to understand the factors that were likely to impact the decision of HCWs to receive the vaccine, especially with the expectation that new strains will continuously evolve and vaccine efficacy may decline—making new vaccine uptake a major contributor to herd immunity over time [10].

Many factors, including widely spread misinformation, can be important contributors, adversely affecting vaccine acceptance [12]. Because HCWs are regarded as a trusted source of information and advice to their patients and acquaintances, it is crucial to highlight their role in spreading awareness and advocating for vaccine acceptance [21]. While various findings have been disseminated regarding the COVID-19 vaccine, factors associated with vaccine refusal have not been studied well among HCWs in Lebanon. Halabi et al. reported a refusal rate of 40% among the Lebanese general population, with female gender, marriage status, and history of vaccine hesitancy being the most influencing factors [22].

Understanding the potential reasons for COVID-19 vaccine refusal among HCWs in Lebanon may help in identifying ways to overcome vaccine gaps in the health care sector and the general population, and improve strategic plans in containing the Severe Acute Respiratory Syndrome-Coronavirus-2 (SARS-CoV-2) [22]. Given the paucity of information about Lebanese HCWs’ acceptance of COVID-19 vaccines and associated demographic factors, we conducted a survey among HCWs from several Lebanese health care institutions to examine COVID-19 vaccine acceptance and identify factors leading to COVID-19 vaccine refusal.

## Methods

### Program Description and Setting

Between January and May 2021, we used selective sampling to conduct a cross-sectional survey using an electronic questionnaire via Qualtrics^®^ (Qualtrics, Provo, UT). HCWs at nine participating hospitals across different provinces in Lebanon were invited as well as other HCWs registered in Orders of Physicians, Nurses, and Pharmacists.

The survey was distributed to HCWs by email, WhatsApp message, or Short Message Service (SMS). The eligibility requirements to participate in the study included being a HCW, 18 years or older, read English or Arabic, and have access to the Internet. Participants had the option to choose English or Arabic. E-consent was required prior to data collection. Our target population included approximately 20,000 physicians, residents, fellows, medical students, nurses (practical and registered nurses), dietitians, dentists, optometrists, psychologists, respiratory therapists, physical therapists, occupational therapists, behavioral therapists, social workers, infection control workers, pharmacists, laboratory and radiology technicians, research assistants and coordinators, and administrative personnel. Based on our previous work and published study [20], and considering the Lebanese HCW population at approximately 40,000 [23], with a vaccine acceptance of 50% and margin of error of 4% (95% CI 46–54%), we calculated a sample size of 592 individuals.

### Measures and Variables

Our survey was redesigned from a previously published work conducted by Malik et al.[20], and included 20 questions (Supplementary Material). Basic demographic information, in addition to information pertaining to participants’ country of origin and religious association were collected. We asked participants to identify factors such as religious barriers, concerns about side effects, lack of trust in vaccine production, and disbelief in vaccine potency that might influence their decision when answering “No” or “Don’t know” about their willingness to receive COVID-19 vaccine once available. Lastly, HCWs were asked about their confidence in organizations and healthcare providers and reliability in media sources as it pertains to disseminating COVID-19 information, with these variables being assigned a value of either 0 or 1 (0 = Very Little/Little/Some/Don’t Know; 1 = Much/Very Much).

The Institutional Review Boards (IRB) at Yale University (IRB protocol number: 2000029237), and at American University of Beirut Medical Center (IRB protocol number: SBS-2020-0563) approved this study, in line with the Code of Ethics of the World Medical Association, the Declaration of Helsinki [24]. After we informed the subjects about the purpose of the study, E-consent was required prior to data collection. Participants were also informed that there will be no risks or direct benefits from their collaboration to this study. The participation was completely voluntary and enrolled subjects retained right to withdraw at any time throughout the study. In addition, to maintain confidentiality, the research team do not have access to their names or contact details. Data were secured on a password protected computer and will only be accessible to the research team members, after which the data will be deleted once the legal retention period expired.

#### Statistical Analysis

Collected data were coded and entered in the software Statistical Package for Social Sciences (SPSS) version 25 (SPSSTM Inc., Chicago, IL United States). Descriptive statistics were conducted to identify the sample demographic characteristics. Moreover, we calculated the frequency and percentage of responses to questions related to COVID-19 vaccine acceptance, reasons for refusal to accept a COVID-19 vaccine, reliability of media sources, and confidence in organizations and healthcare providers.

Finally, univariate analyses were conducted. The explanatory variables were first tested 1 by 1 against the dependent variable for the presence of a significant association using the binomial logistic regression. In the multivariable logistic regression model, we included variables reported in the literature to be associated with COVID-19 vaccine acceptance considering them as potential confounders, such as age, gender, religious association, and educational level [11, 14, 20, 25–27], and variables that showed a significant association at *p* ≤ 0.05 across any categories in the univariate analysis. The goodness-of-fit statistic is reported to determine if the model provides a good fit for the data (*p* > 0.05) [28]. The strength of association was interpreted using the adjusted odds ratio (AOR) with 95% confidence Interval (CI). A p ≤ 0.05 was considered statistically significant.

## Results

### Sample Characteristics

Among the 879 HCWs who completed the survey, the majority were Lebanese (*n* = 858; 97.6%), between 25 and 34 years old (*n* = 301; 34.2%), female (*n* = 619; 70.4%), Muslim (*n* = 423; 48.1%), had a graduate/professional degree (*n* = 492; 56%), and had no chronic disease (*n* = 708; 80.5%). Nurses (*n* = 385, 45.5%) accounted for the largest group among respondents. Table 1 shows the characteristics of the survey participants.

**Table 1:** Baseline characteristics of the study population

Variables	Frequency (*N*)	Percentage (%)
Age (*n* = 879)		
18–24 years old	69	7.8
25–34 years old	301	34.2
35–44 years old	282	32.1
45–54 years old	127	14.4
55 + years	100	11.4
Gender (*n* = 879)		
Male	254	28.9
Female	619	70.4
Other	6	0.7
Nationality (*n* = 879)		
Lebanese	858	97.6
Non-Lebanese*	21	2.4
Religion (*n* = 879)		
Christian	328	37.3
Muslim	423	48.1
None	24	2.7
Other not specified	21	2.4
Prefer not to answer	83	9.4
Chronic diseases (*n* = 879)		
No	708	80.5
Yes	162	18.4
Unknown	9	1.0
Educational level (*n* = 879)		
Less than high school	4	0.5
High school	53	6.0
College	330	37.5
Graduate/Professional	492	56.0
Profession (*n* = 846)		
Registered nurses	385	45.5
Physicians	192	22.7
Administrators	108	12.8
Practical nurses	29	3.4
Laboratory personnel	28	3.3
Pharmacists	20	2.4
Radiology personnel	10	1.2
Infection control personnel	11	1.3
Research assistants and coordinators	10	1.2
Other**	53	6.2

*Non-Lebanese: Egyptian, Jordanian, Palestinian, Philippine, Syrian, Other not specified

**Other: Psychologists, physical therapists, respiratory therapists, dietitians, optometrists, occupational therapists, social workers, dental hygienists, public health inspectors

### COVID-19 Vaccine Acceptance and Demographic Characteristics

**Fig. 1:**
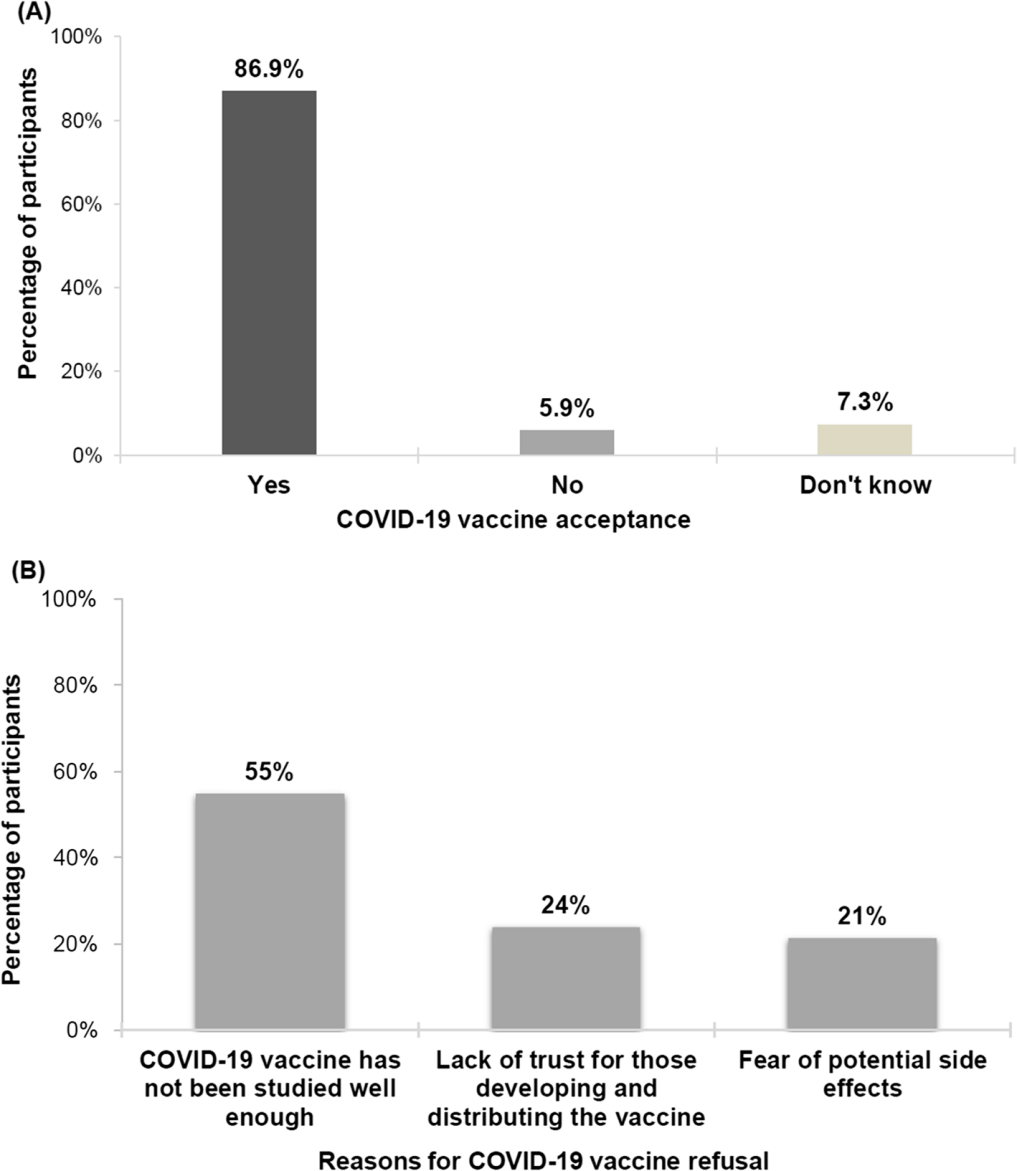
**A** Rate of COVID-19 vaccine acceptance among HCWs in Lebanon. **B** Reasons for COVID-19 vaccine refusal among HCWs in Lebanon

Among the 879 participants, 762 (86.8%) were willing to receive the COVID-19 vaccine, 52 (5.9%) refused, 64 (7.3%) were undecided whether to receive it or not, and 1 (0.1%) did not answer the question on whether they would take the vaccine or not (Fig. 1A). Of the 116 (13.2%) who said they would not accept a COVID-19 vaccine, 62 (53.4%) reported that the vaccine had not been studied well enough, 27 (23.3%) reported lack of trust of those developing and distributing the vaccine, 24 (20.7%) reported fear of potential side effects, and 3 (2.6%) did not answer the question related to why they would not take the vaccine (Fig. 1B). COVID-19 vaccine acceptance rates were shown to differ among the various demographic groups. Males (226/254, 88.9%), those aged 55 or older (95/100, 95%), and Lebanese (745/857, 86.9%) had the highest rates of acceptance (Table 2).

**Table 2:** COVID-19 vaccine acceptance rates

Characteristics	COVID-19 Vaccine acceptance, *N* (%)			
	Overall	Yes	No	Unknown
Gender (*N* = 878)				
Male	254	226 (89.0)	14 (5.5)	14 (5.5)
Female	618	532 (86.1)	37 (6.0)	49 (7.9)
Other	6	4 (66.7)	1 (16.7)	1 (16.7)
Age (*N* = 878)				
18–24 years old	69	59 (85.5)	5 (7.2)	5 (7.2)
25–34 years old	301	256 (85.0)	21 (7.0)	24 (8.0)
35–44 years old	282	235 (83.3)	22 (7.8)	25 (8.9)
45–54 years old	126	117 (92.9)	3 (2.4)	6 (4.8)
55 + years	100	95 (95.0)	1 (1.0)	4 (4.0)
Religion (*N* = 878)				
Christian	328	296 (90.2)	11 (3.4)	21 (6.4)
Muslim	422	356 (84.4)	34 (8.1)	32 (7.6)
None	24	22 (91.7)	1 (4.2)	1 (4.2)
Other	21	19 (90.5)	1 (4.8)	1 (4.8)
Prefer not to answer	83	69 (83.1)	5 (6.0)	9 (10.8)
Nationality (*N* = 878)				
Lebanese	857	745 (86.9)	50 (5.8)	62 (7.2)
Non-Lebanese	21	17 (81.0)	2 (9.5)	2 (9.5)
Chronic diseases (*n* = 869)				
No	707	610 (86.3)	46 (6.5)	51 (7.2)
Yes	162	144 (88.9)	5 (3.1)	13 (8.0)
Educational level (*n* = 878)				
Less than high school	4	4 (100)	0 (0.0)	0 (0.0)
High school	53	44 (83.0)	7 (13.2)	2 (3.8)
College	329	277 (84.2)	19 (5.8)	33 (10.0)
Graduate/Professional	492	437 (88.8)	26 (5.3)	29 (5.9)
Profession (*n* = 845)				
Registered nurses	385	332 (86.2)	23 (6.0)	30 (7.8)
Physicians	192	181 (94.3)	5 (2.6)	6 (3.1)
Administrators	107	88 (82.2)	7 (6.5)	12 (11.2)
Practical nurses	29	21 (72.4)	6 (20.7)	2 (6.9)
Laboratory personnel	28	19 (67.9)	4 (14.3)	5 (17.9)
Pharmacists	20	18 (90.0)	0 (0.0)	2 (10.0)
Radiology personnel	10	9 (90.0)	0 (0.0)	1 (10.0)
Infection control personnel	11	11 (100)	0 (0.0)	0 (0.0)
Research assistants and coordinators	10	9 (90.0)	0 (0.0)	1 (10.0)
Otherǂ	53	49 (92.5)	2 (3.8)	2 (3.8)
Previous SARS-COV2 infection (*n* = 878)				
No	586	532 (90.8)	28 (4.8)	26 (4.4)
Yes	259	207 (79.9)	19 (7.3)	33 (12.7)
Don't know	33	23 (69.7)	5 (15.2)	5 (15.2)
Previous SARS-COV2 infection of someone in the immediate social network (*n* = 878)				
Yes	719	623 (86.6)	47 (6.5)	49 (6.8)
No	137	121 (88.3)	4 (2.9)	12 (8.8)
Don't know	22	18 (81.8)	1 (4.5)	3 (13.6)
Knowledge level about the novel coronavirus/COVID-19 (*n* = 878)				
Very poor	2	0 (0.0)	2 (100)	0 (0.0)
Poor	14	8 (57.1)	3 (21.4)	3 (21.4)
Average	104	89 (85.6)	4 (3.8)	11 (10.6)
Good	351	300 (85.8)	19 (5.4)	32 (9.1)
Very good	405	364 (89.9)	24 (5.9)	17 (4.2)
Don't know	2	1 (50.0)	0 (0.0)	1 (50.0)

^¶^Non-Lebanese: Egyptian, Jordanian, Palestinian, Philippine, Syrian, Other not specified

ǂOther: Psychologists, physical therapists, respiratory therapists, dieticians, optometrists, occupational therapists, social workers, dental hygienists, public health inspectors

The multivariable logistic regression analysis showed that after adjusting for gender, age, religion, and education level, significant findings were seen in a couple of demographic factors (Table 3). Participants with a previous SARS-CoV-2 infection (OR: 0.47; 95% CI 0.30–0.75; *p* = 0.002) and those who did not know if they had a previous infection (OR: 0.24; 95% CI 0.10–0.60; *p* = 0.002) were less likely to accept the vaccine compared to those with no previous SARS-CoV-2 infection. No significant association was seen in the other demographic factors after adjustment.

**Table 3:** Association of demographic, social, and behavioral characteristics with COVID-19 vaccine acceptance using univariate and multivariable logistic regression analyses

	COVID-19 Vaccine acceptance (No or Don't know / Yes)			
Characteristics	Unadjusted OR [95% CI]	*p* value	Adjusted OR [95% CI]	*p* value
Gender (*N* = 878)				
Male	Ref		Ref	
Female	0.766 [0.487–1.207]	0.25	0.74 [0.44–1.24]	0.26
Other	0.248 [0.043–1.415]	0.12	0.34 [0.38–3.08]	0.34
Age (*N* = 878)				
18–24 years old	0.311 [0.101–0.953]	**0.04**	0.57 [0.17–1.92]	0.37
25–34 years old	0.299 [0.115–0.777]	**0.01**	0.51 [0.19–1.38]	0.19
35–44 years old	0.263 [0.102–0.682]	**0.006**	0.478 [0.17–1.29]	0.15
45–54 years old	0.684 [0.222–2.110]	0.50	1.11 [0.33–3.73]	0.86
55 + years	Ref		Ref	
Religion (*N* = 878)				
Christian	Ref		Ref	
Muslim	0.583 [0.372–0.914]	**0.02**	0.79 [0.47–1.32]	0.38
None	1.189 [0.267–5.291]	0.82	1.83 [0.26–12.68]	0.54
Other	1.027 [0.229–4.612]	0.97	1.45 [0.29–7.19]	0.65
Prefer not to answer	0.533 [0.270–1.052]	0.07	0.62 [0.29–1.33]	0.23
Nationality (*N* = 878)				
Lebanese	Ref			
Non-Lebanese^¶^	0.639 [0.211–1.933]	0.43		
Chronic diseases (*n* = 869)				
No	Ref			
Yes	1.272 [0.745–2.172]	0.38		
Educational level (*n* = 878)				
Less than high school/High school	Ref		Ref	
College	0.999 [0.462–2.160]	0.99	0.53 [0.17–1.64]	0.28
Graduate/Professional	1.490 [0.693–3.202]	0.31	0.49 [0.15–1.58]	0.24
Profession (*n* = 845)				
Registered nurses	Ref		Ref	
Physicians	2.627 [1.339–5.155]	**0.005**	1.94 [0.91–4.14]	0.08
Administrators	0.739 [0.416–1.313]	0.30	0.73 [0.39–1.34]	0.31
Practical nurses	0.419 [0.177–0.995]	**0.049**	0.36 [0.11–1.19]	0.09
Laboratory personnel	0.337 [0.145–0.784]	**0.01**	0.36 [0.14–0.91]	0.03
Pharmacists	1.437 [0.324–6.371]	0.63	1.27 [0.27–6.00]	0.75
Other professions^ǂ^	2.075 [0.861–5.001]	0.10	1.84 [0.73–4.61]	0.19
Previous SARS-COV2 infection (*n* = 878)				
No	Ref		Ref	
Yes	0.404 [0.267–0.611]	< 0.001	0.47 [0.30–0.75]	**0.002**
Don't know	0.233 [0.106–0.516]	< 0.001	0.24 [0.10–0.60]	**0.002**
Previous SARS-COV2 infection of someone in the immediate social network (*n* = 878)				
No	Ref			
Yes	0.858 [0.488–1.508]	0.60	1.16 [0.62–2.18]	0.64
Don't know	0.595 [0.179–1.980]	0.40	1.13 [0.32–3.92]	0.85
Knowledge level about the novel coronavirus/COVID-19 (*n* = 878)				
Don't know/Very poor/Poor	Ref		Ref	
Good/Very Good	1.768 [1.076–2.905]	**0.02**	1.3 [0.7–2.4]	0.32

*OR* Odds Ratio, *CI* Confidence Interval, *n* Frequency, *Ref* Reference

^¶^Non-Lebanese: Egyptian, Jordanian, Palestinian, Philippine, Syrian, Other not specified

^ǂ^Other professions: Infection control personnel, radiology personnel, research personnel, psychologists, physical therapists, respiratory therapists, dieticians, optometrists, occupational therapists, social workers, dental hygienists, public health inspectors

The bold values indicate significant *p*-values ≤0.05

### Reliability of Media Sources and Confidence in Organizations pertaining to COVID-19 Information

Participants reported the WHO (*n* = 609; 69.3%), healthcare providers (*n* = 598; 68.0%), and health officials (*n* = 491; 55.9%) as the most reliable media sources of COVID-19 information. Additionally, participants reported the highest confidence in healthcare providers (*n* = 611 69.5%) and the WHO (*n* = 567; 64.5%).

### Reliability of Media Sources and Confidence in Organizations and Healthcare Providers pertaining to COVID-19 Information across the COVID-19 Vaccine Acceptance Landscape

**Fig. 2:**
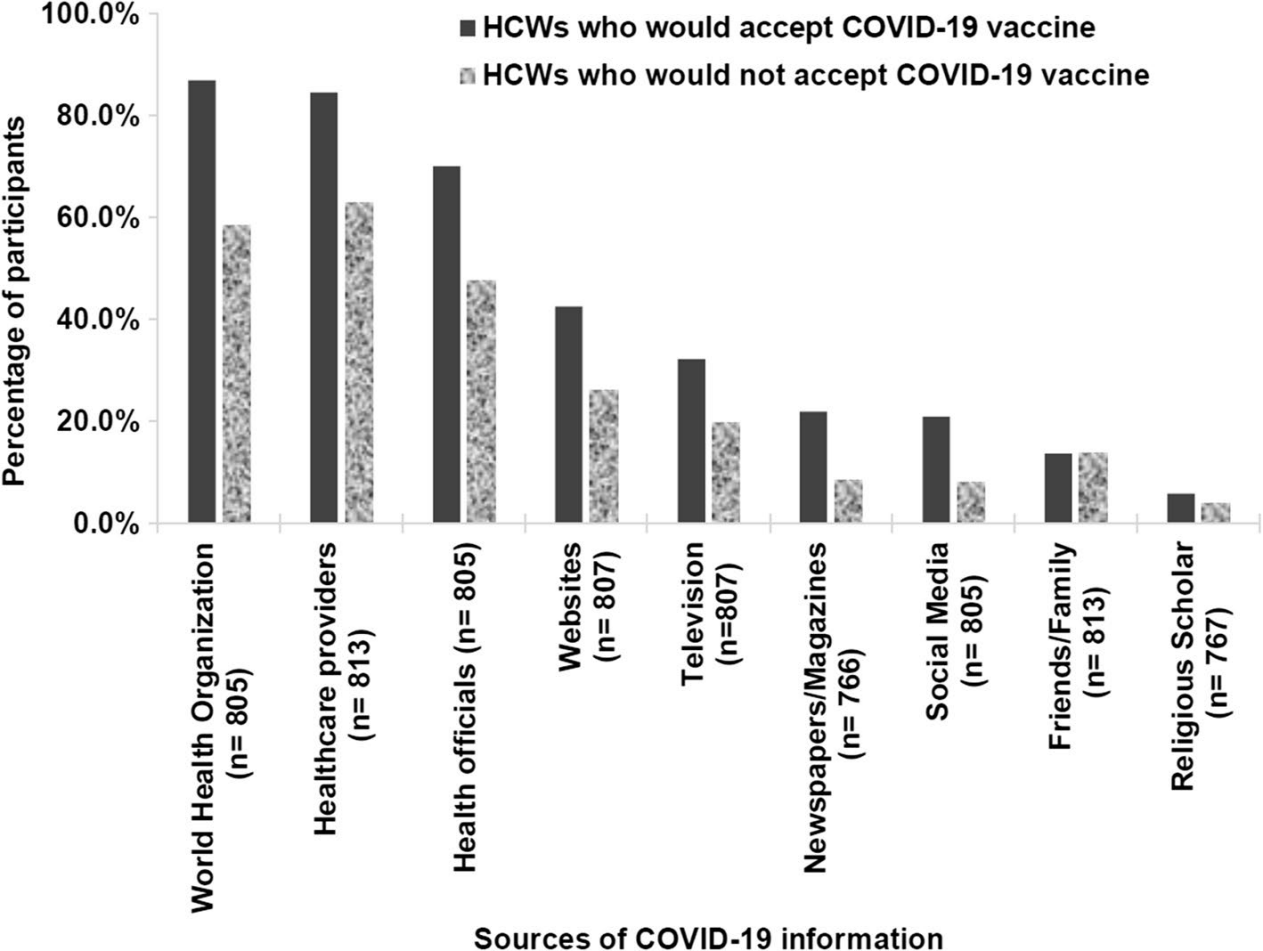
Reliability of COVID-19 sources of information among HCWs

Participants who would take the COVID-19 vaccine (*n* = 762; 86.8%) reported the WHO (*n* = 609; 86.8%), healthcare providers (*n* = 598; 84.5%), health officials (*n* = 491; 69.9%) as the most reliable media sources of COVID-19 information. Participants who would not take the COVID-19 vaccine (*n* = 116; 13.2%) reported healthcare providers (*n* = 66; 62.9%), the WHO (*n* = 60; 58.3%), and health officials (*n* = 49; 47.6%) as the most reliable sources of COVID-19 information (Fig. 2).

Additionally, participants who would take the COVID-19 vaccine (*n* = 762; 86.8%) reported the highest confidence in healthcare providers (*n* = 611; 85.9%), the WHO (= 567; 80.8%), and the health ministry (*n* = 400; 56.6%). Participants who would not take the COVID-19 vaccine (*n* = 116; 13.2%) reported the highest confidence in healthcare providers (*n* = 64; 61.0%), the WHO (*n* = 52; 50.5%) and the health ministry (*n* = 39; 37.1%).

## Discussion

During early 2021, the majority of HCWs in Lebanon reported they would accept a COVID-19 vaccine (86.8%). Our finding of 86.8% is significantly higher than what was previously reported among Lebanese HCWs (26.8%) [29]. The major difference between both acceptance rates may be because our study collected data more recently (January 2021 to May 2021), compared to the previous study that collected data only in January 2021. Therefore, the acceptance of COVID-19 vaccines among Lebanese HCWs may have been higher in our study as evidence for the efficacy of the vaccines became more widely available over time [30]. Another possible reason for the major difference in acceptance rates may be due to the difference in methodologies between both studies—our study distributed the survey via email, WhatsApp message, or Short Message Service (SMS), while the previous study only used social media platforms to recruit participants. Moreover, our high acceptance rate surpasses that of previous studies conducted in HCWs in the KSA (64.9%), the United Arab Emirates (UAE) (89.2%), France (76.9%), Belgium (76.0%), Malta (52%), U.S. (36%), and the Democratic Republic of Congo (27.7%) [14, 19, 25, 31, 32]. While this is not a direct comparison as some of these countries were surveyed prior to the vaccine becoming available, some countries, such as the UAE (89.2%), have reported a similarly high acceptance rate as found in our study.

Among those who refused COVID-19 vaccination, the main reason for their refusal was that the vaccine had not been tested well yet (54.9%). The rapid development of COVID-19 vaccines is thus manifesting as a major factor in vaccine hesitancy, as studies from the U.S. imply that the accelerated vaccine approval process played an important role in this regard [33]. Additionally, other reasons for vaccine refusal in our survey were attributed to lack of trust in those developing and distributing the vaccine as well as fear of potential side effects. Lack of trust is a global phenomenon as the amount of circulating misinformation about the SARS-CoV-2 virus has been a major factor in people losing confidence in governments and health care officials [34]. This offers an opportunity to address the important role of government officials and the Ministry of Public Health in increasing vaccine confidence by disseminating accurate information about the COVID-19 vaccine, whether through expert panels on the science and manufacturing of these vaccines, or the continued showcase of the incredibly abundant emerging safety data.

The vaccines approved and administered in Lebanon are currently the Pfizer-BioNTech, Oxford-AstraZeneca, Sputnik V, and Sinopharm vaccines [7]. Although these vaccines have been thoroughly tested, approved, and administered to hundreds of millions around the world, the high acceptance rate reported in HCWs in this study was greater than that of the general population, which was found in a previous cross-sectional study conducted in Lebanon between November and December 2020 (21.4%) [22]. This may be because both studies were conducted at different time points—the general population was surveyed before COVID-19 vaccines were introduced in Lebanon, while our study was conducted when vaccine administration began for HCWs. Additionally, HCWs may have a higher COVID-19 vaccine acceptance rate than the general population because they are likely to be more knowledgeable of the COVID-19 pandemic, given their direct exposure to COVID-19 cases.

Studies have shown that the recommendation of governments and healthcare providers of the COVID-19 vaccination is a positive predictor of acceptance in patients [35]. The disparity in acceptance between HCWs and the general population offers an opportunity for our health care professionals to mobilize the population to encourage individuals to get the COVID-19 vaccine. This should be done through campaigns, media appearances, and active encouragement in clinics and hospital settings to boost receipt as much as possible. This suggests that the Lebanese MOPH could also work with the Lebanese HCWs to develop COVID-19 messaging and educational campaigns that cater to the general Lebanese population. One intervention that has worked well to help increase COVID-19 vaccine rates is the Inter-ministerial and Municipal Platform for Assessment Coordination and Tracking (IMPACT) national vaccination platform [36]. IMPACT generates online real-time data dashboards, supports assisted vaccine registration, allows users to report adverse events following vaccination, and includes a call center that assists with rescheduling appointments [36]. Overall, IMPACT has increased the transparency of the Lebanese MOPH’s vaccination rollout and boosted public trust [36].

As stated earlier, a major pillar in vaccine hesitancy is related to reduced confidence in vaccine manufacturers, distributors, governments and international organizations. Our study found that a majority of Lebanese HCWs highly regard the Lebanese MOPH and the WHO as reliable sources of information in relation to COVID-19. Additionally, Lebanese HCWs reported confidence in health care providers (doctors, nurses, and pharmacists), which underscores the importance of spreading the correct scientific information by these authoritative bodies and successfully competing with widespread misinformation falsely spread on social media. Reported confidence and trust of HCWs in their peers also highlights the need for healthcare providers to have peer-based interventions (e.g., engaging in expert panels and conferences to learn about the evolving vaccine safety and effectiveness data), which may increase COVID-19 vaccine acceptance [37].

After adjusting our data for multiple demographic characteristics, no significant differences in vaccine acceptance were found according to age or gender of participants. This is in contrast to multiple other studies that found women HCWs to be less likely to accept the COVID-19 vaccine [14, 26]. The fact that the largest number of health care professionals surveyed were nurses and considering that the Lebanese nurse force is mainly female predominant, this might offer a possible explanation [38]. Nurses, since the beginning of the COVID-19 pandemic, have been spearheading the COVID-19 response and are thus responsible for taking care of the sickest and critical patients. This could explain the high rates of vaccine approval among this demographic, who may fear the morbidity and mortality associated with the COVID-19 infection.

Similarly, after adjustment of multiple demographic factors, no significant findings concerning the impact of religion on willingness to take the vaccine were reported in our study. When comparing with other studies, no clear association between religion and vaccine hesitancy was seen, as some studies showed religion influencing vaccine acceptance, while other studies showed no significant effects [39–42]. Our finding that there was no significant association between chronic conditions and vaccine acceptance was similar to a study conducted in Singapore [43], yet different from a separate study conducted in Ethiopia [44]. The lack of significant association between chronic conditions and vaccine acceptance found in our study may be due to HCWs’ low risk perception for chronic conditions as being a catalyst for increasing the risk of hospitalization and death. As for religion, given Lebanon’s religious diversity among its population, it would be interesting to further investigate participants’ religious views and their level of religiosity to understand this lack of association.

HCWs with a previous SARS-CoV-2 infection were found to be less likely to take the COVID-19 vaccine as compared with those who had not been infected. A possible explanation of this finding is a false perception of acquired natural immunity in these individuals prompting them to decline future vaccines. According to the Center for Disease Control and Prevention (CDC) in the U.S., current recommendations are for individuals to be vaccinated regardless of previous infection status, as we do not yet know the extent and duration of naturally produced immunity from COVID-19 [45]. This subgroup of Lebanese HCWs must thus be targeted by pro-vaccine campaigns, and health care officials must take the lead in correcting any misinformation in this regard, especially before it begins to spread across the general Lebanese population. The uncertainty of one’s SARS-Cov-2 infection history may influence an individual’s willingness to take the vaccine. Those who may have caught the virus and have acquired immunity against it may decide that they do not need the vaccine. Others may claim that they may have contracted the virus, yet still decide to take the vaccine for extra precaution and prevention of an additional future infection.

Moreover, because several SARS-CoV-2 variants emerged since we collected our data in the beginning of 2021, HCWs' perceptions may have changed during this time. For example, Omicron variants that started in January 2022 had a significant impact on HCWs, where only two-thirds of HCWs felt that vaccination was the best option to prevent the spread of the Omicron variant from early 2022—indicating the need for further messaging campaigns for COVID-19 vaccination [46].

### Strengths and Limitations

While our study included a large enough sample size of 879 HCWs, and we knew our target population included approximately 20,000 HCWs, we were unable to track the number of emails and messages (WhatsApp and SMS) that were received, returned, and/or went to spam. Therefore, we were unable to calculate the response rate. Also, given that we used a purposive sampling method and not a stratified random sample; our findings may not be generalizable to the whole Lebanese HCW population. Furthermore, our study examined the intent to vaccinate, which may not always translate into vaccine uptake [47]. Moreover, because our survey did not define “chronic conditions” and whether these conditions were related to COVID-19, participants may have interpreted this differently, which may have affected our results.

Given that the French language is one of the most commonly spoken languages in Lebanon and the survey was only provided in English or Arabic, participants who did not read and understand English or Arabic may have not participated in our study. Lastly, our findings may be influenced by a social desirability bias, as HCWs may respond to the survey questions in a manner that is viewed favorably by others.

However, the main strength of this study is that participants were from multiple hospitals throughout different areas of Lebanon, so it is representative in this regard. Also, to our knowledge, this is the first study in Lebanon that helps understand COVID-19 vaccine perception and acceptance among HCWs, as opposed to studies done on the general population.

## Conclusion

Our study showed that 86.8% of the Lebanese HCWs would accept a COVID-19 vaccine. Refusal of vaccination was mainly due to the perception that COVID-19 vaccines have not been tested well. The surveyed HCWs showed great confidence in the MOPH and WHO, which must be key communication platforms in the COVID-19 vaccination campaign. Lebanese health care authorities can utilize these findings to establish COVID-19 programs to improve vaccine acceptance and uptake among HCWs and the general public.

## Supplementary Information

Below is the link to the electronic supplementary material.

Supplementary file1 (DOCX 32 KB)

## Data Availability

Data are available upon request due to privacy and ethical restrictions.

## Abbreviations

HCWs: Health care workers
COVID-19: Coronavirus Disease-2019
LMICs: Low- and middle-income countries
COVAX: COVID-19 Vaccines Global Access
WHO: World Health Organization
MOPH: Ministry of Public Health
mRNA: Messenger Ribonucleic acid
U.S.: United States
KSA: Kingdom of Saudi Arabia
UN: United Nations
SARS-CoV-2: Severe Acute Respiratory Syndrome-Coronavirus-2
IRB: Institutional Review Board
SMS: Short Message Service
SPSS: Statistical Package for Social Sciences
OR: Odds Ratio
CI: Confidence Interval
UAE: United Arab Emirates
CDC: Center for Disease Control and Prevention
