# Supplementary material for: COVID-19 Vaccine Acceptance and Hesitancy Among Health Care Workers in Lebanon
Source: J Epidemiol Glob Health. 2023 Feb 3;13(1):55–66. doi: 10.1007/s44197-023-00086-4 (PMC9896451; doi:10.1007/s44197-023-00086-4)
Supplement: Supplementary file 1 — Supplementary file1 (DOCX 32 KB) [file 44197_2023_86_MOESM1_ESM.docx]

**COVID-19 Vaccine Acceptance and Hesitancy among Health Care Workers in Lebanon**

**Journal of Epidemiology and Global Health**

**Nour J. Youssef, MD^a,b^, Nadim K. Tfaily, MD^a,1^, Mohammad Bahij M. Moumneh, BS^a,1^ , Celina F. Boutros, BSN, MPH^a^,** Jad A. Elharake, MPH^c,d^, Amyn A. Malik, MBBS, MPH, PhD^c,d^, SarahAnn M. McFadden, PhD, RN, CPN^c,d^, Bayan Galal^e^, Inci Yildirim, MD, PhD, MSc^c,d^, Kaveh Khoshnood, PhD^f^, Saad B. Omer, MBBS, MPH, PhD, FIDSA^c,d,f,g^, Ziad A. Memish, MD, FACP, FRCPC, FRCPE, FIDSA^h^**^,i,j^ , Ghassan S. Dbaibo, MD,** FAAP**^a,b,*^.**

***Corresponding Author:** Ghassan S. Dbaibo, MD, FAAP, Center for Infectious Diseases Research, Division of Pediatric Infectious Diseases, Department of Pediatrics and Adolescent Medicine, American University of Beirut Medical Center, Hamra, Beirut 1107, Lebanon ([gdbaibo@aub.edu.lb](mailto:gdbaibo@aub.edu.lb))

**Supplementary Material: English version of the survey.**

Q1 What is your gender? Please select the answer of your choice.

- Male
- Female
- Other

Q2 What is your age? Please select the answer of your choice.

- 18 - 24 years
- 25 - 34 years
- 35 - 44 years
- 45 - 54 years
- 55+ years

Q3 What is your country of origin? Please select the answer of your choice.

- Egypt
- India
- Jordan
- Lebanon
- Palestine
- Iraq
- Philippines
- Saudi Arabia
- Sudan
- Syria
- Other

Q4 What is your religion? Please select the answer of your choice.

- Christianity
- Islam
- None
- Other
- Prefer not to answer

Q5 What is your level of education? Please select the answer of your choice.

- Less than high school
- High school
- College
- Graduate/Professional

Q6 Do you work in healthcare?

- Yes.
- No.
- Don't know.

Q7 Which category of healthcare worker do you belong to?

- Physician
- Dentist
- Registered nurse
- Practical nurse
- Pharmacist
- Psychologist
- Physical therapist
- Respiratory therapist
- Dietician
- Optometrist
- Laboratory technician/Doctor
- Radiology technician
- Behavioral therapist
- Occupational therapist
- Social worker
- Infection control worker
- Research assistant or coordinator
- Administrator, clerk, or secretary
- Other (e.g., dental hygienist or public health inspector)

Q8 Do you have a chronic illness?

- Yes.
- No.
- Don't know.

Q9 Have you been sick with novel coronavirus/COVID-19?

- Yes, tested.
- Yes, but not yet tested.
- No.
- Don't know.

Q10 Do you know in your immediate social network anyone sick with novel coronavirus/COVID-19?

- Yes.
- No.
- Don't know.

Q11 How would you rate your knowledge level about the novel coronavirus/COVID-19?

- Very poor
- Poor
- Average
- Good
- Very Good
- Don't know

Q12 Which of the following is correct about the definition of novel coronavirus/COVID-19?

- Novel coronavirus/COVID-19 is a respiratory disease caused by a viral infection.
- Novel coronavirus/COVID-19 is not contagious.
- Novel coronavirus/COVID-19 never leads to death.
- Don’t know.

Q13 Which of the following is correct about transmission route of novel coronavirus/COVID-19?

- Novel coronavirus/COVID-19 is transmitted through coughing or sneezing.
- Novel coronavirus/COVID-19 is not transmitted by close contact with people.
- Don’t know.

Q14 Which of the following is correct about “close contact” for the novel coronavirus/COVID-19?

- “Close contact” involves physical contact.
- Relatives and healthcare workers are excluded from the category of close contact.
- Don’t know.

Q15 If a vaccine for COVID-19 becomes available in your country and is recommended for you, will you take it?

- Yes.
- No.
- Don't know.

Q16 What is the main reason why you would not get the vaccine?

- Religion.
- Fear of potential side effects.
- Lack of trust for those creating and distributing the vaccine.
- Do not believe vaccines work.
- COVID-19 vaccine has not been studied well enough.
- There is a microchip in the vaccine that will track and control me.

Q17 Which of the following are effective preventative measures for yourself and/or others against the novel coronavirus?

|  | Yes | No |
| --- | --- | --- |
| Avoided travel |  |  |
| Hand washing |  |  |
| Using disinfectants |  |  |
| Avoiding touching your eyes, nose, and mouth with unwashed hands |  |  |
| Avoided eating at a restaurant |  |  |
| Staying home when you are sick |  |  |
| Covering your cough or sneeze |  |  |
| Avoiding close contact with someone who is sick |  |  |
| Taking herbal supplements |  |  |
| Exercising regularly |  |  |
| Eating a balanced diet |  |  |
| Taking supplements (Vitamin C, Zinc, Selenium, etc.) |  |  |
| Using caution when receiving any letter or delivery |  |  |
| Avoiding eating meat |  |  |
| Wearing a face mask |  |  |
| Using hand sanitizer |  |  |
| Social/physical distancing |  |  |

Q18 For the following sources of information in Lebanon, please rate how reliable you feel they are with respect to the novel coronavirus.

|  | Very Little | Little | Some | Much | Very Much | Don't know |
| --- | --- | --- | --- | --- | --- | --- |
| Television |  |  |  |  |  |  |
| Newspapers/Magazines |  |  |  |  |  |  |
| Websites |  |  |  |  |  |  |
| Friends/Family |  |  |  |  |  |  |
| Healthcare providers (ex: doctors, nurses, paramedics, and pharmacists) |  |  |  |  |  |  |
| Health Officials (ex: health ministry) |  |  |  |  |  |  |
| Social Media |  |  |  |  |  |  |
| Religious Scholar |  |  |  |  |  |  |
| World Health Organization (WHO) |  |  |  |  |  |  |

Q19 How much confidence do you have in each of these organizations in Lebanon?

|  | Very Little | Little | Some | Much | Very Much | Don't Know |
| --- | --- | --- | --- | --- | --- | --- |
| Healthcare providers (ex: doctors, nurses, paramedics, and pharmacists) |  |  |  |  |  |  |
| Health Ministry |  |  |  |  |  |  |
| Government |  |  |  |  |  |  |
| Religious Scholar |  |  |  |  |  |  |
| World Health Organization (WHO) |  |  |  |  |  |  |

Q20 Have your working hours been cut because of the current COVID-19 pandemic?

- Yes.
- No.
